# Correspondence on “Highly Volatile POPs in Urban Air across Asia and Africa: Dominance of Volatile Methylsiloxanes”

**DOI:** 10.1021/acs.est.6c02638

**Published:** 2026-04-24

**Authors:** Christopher E. Brunet, Saeideh Mohammadi, Behrooz Roozitalab, Nora K. Gibson, Rafael P. Fernandez, Alfonso Saiz-Lopez, Keri C. Hornbuckle, Charles O. Stanier

**Affiliations:** † Department of Civil and Environmental Engineering, 4083 University of Iowa, Iowa City, Iowa 52242, United States; ‡ IIHR-Hydroscience and Engineering, 4083 University of Iowa, Iowa City, Iowa 52242, United States; § Department of Chemical and Biochemical Engineering, 4083 University of Iowa, Iowa City, Iowa 52242, United States; ∥ 53593 NSF National Center for Atmospheric Research, Boulder, Colorado 80301, United States; ⊥ Institute for Interdisciplinary Science (ICB), National Research Council (CONICET), 63019 FCEN-UNCuyo, Mendoza 5501, Argentina; # Department of Atmospheric Chemistry and Climate, Institute of Physical Chemistry Blas Cabrera, 69568 CSIC, Madrid 28006, Spain

We read with great interest the recent article by Xiao et al. that presented persistent volatile organic compound measurements across six major cities in Asia and Africa.^1^ We were particularly interested in their measurements of volatile methyl siloxanes (VMS), which have rarely or never been reported in many of the studied regions (Figure 1). These new measurements provide an opportunity to perform comparisons to the novel global VMS model described in Brunet et al.^2^ Here, we explore these comparisons to evaluate emission factors and model representation in these previously understudied locations.

**Figure 1:**
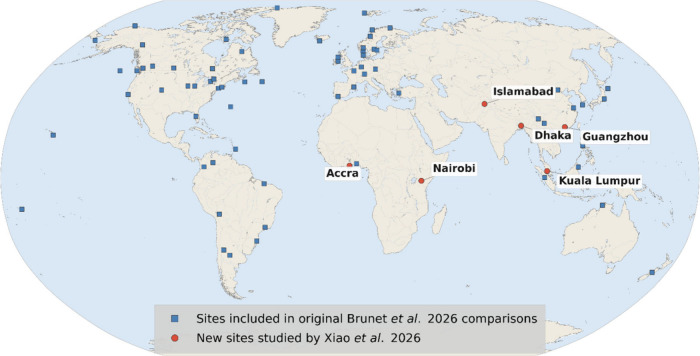
Study sites where ambient atmospheric VMS have been reported in the literature. Blue boxes indicate sites from the original study by Brunet et al. Red dots indicate the sites of Xiao et al. where comparisons described here were performed.

In the work of Brunet et al., atmospheric VMS concentrations were simulated within the short-lived halogen variant of the Community Earth System Model (CESM2-SLH) with bottom-up emissions based on population density and country-level per-capita personal care product (PCP) consumption.^2,3^ Our model shows reasonably good agreement with reported VMS measurements, but also enables comparisons to new measurements as they become available. Here, we compare surface concentrations of D4–D6 predicted by our model to the new measurements of Xiao et al. (Figure 2). These comparisons further support the findings of both studies, underscoring the need for improved industrial VMS emissions inventories.

**Figure 2:**
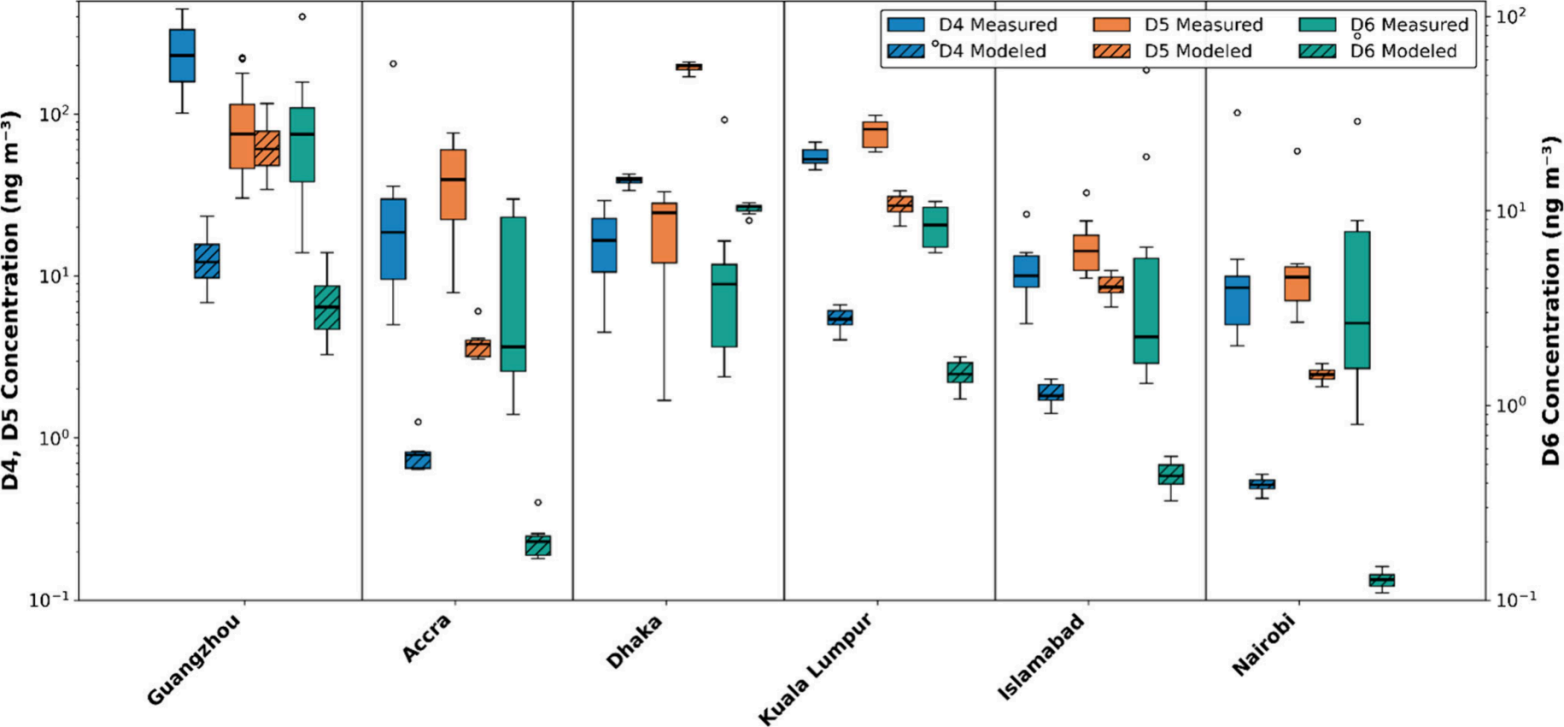
Comparison of model-predicted (Brunet et al.) and measured (Xiao et al.) surface concentrations of D4–D6 across six cities. Note that data for D4 and D5 are plotted on the left axis while data for D6 are plotted on the right axis; both axes are on a log scale.

Xiao et al. provided compelling evidence that D4 emissions in Guangzhou were driven primarily by industrial releases. This is supported by the fact that model-predicted D4 concentrations (based on a PCP-only emissions scheme) in Guangzhou (mean of 13 ng m^–3^) were an order of magnitude lower than the reported measurements (mean of 290 ng m^–3^) (Figure 2). This discrepancy effectively isolates the industrial D4 signal and points to the need for emissions inventories accounting for these industrial releases. However, good agreement between measured and predicted D5 (90 ng m^–3^ vs 65 ng m^–3^) suggests that PCP use likely remains the primary D5 source in Guangzhou (Figure 2), demonstrating that the model can provide robust D5 predictions even in areas with industrial emissions.

Measured concentrations and concentration spreads of D4–D6 were underpredicted in Accra, Kuala Lumpur, Islamabad, and Nairobi (Figure 2). These discrepancies are consistent with several nonexclusive factors: spatial resolution limitations where the coarse model grid dilutes peak urban concentrations, a lack of spatiotemporal variation in modeled emissions, underestimated national emission factors in rapidly developing economies, and urban–rural contrasts in PCP usage. For example, the large Accra discrepancy (mean D5, 40 ng m^–3^ (measured) vs 3.9 ng m^–3^ (modeled)) may reflect subnational variability in PCP use not captured by country-average consumption factors. While urban–rural differences in per-capita PCP consumption are modest in North America and Europe, urban populations in several countries studied by Xiao et al. may consume PCP at substantially higher rates than national averages imply. Such disparities have been documented in both African and Asian countries.^4–6^ Furthermore, Cheng et al. reported mobile VMS measurements suggesting D5 concentrations in China may be driven by GDP in addition to population density.^7^ While overestimation of atmospheric degradation rates could also contribute to model underprediction, these measurement sites are predominantly source-dominated urban environments where ambient concentrations are largely insensitive to removal rates, particularly for species with long atmospheric lifetimes like VMS. Additional geographically distributed measurements would be invaluable for discriminating among these hypotheses.

Model overprediction in Dhaka, Bangladesh, may be a result of challenges in simulating boundary layer dynamics in the Indo-Gangetic Plain due to local aerosol pollution or an overestimation of the city’s VMS emission rate related to GDP or other factors.^8^ However, we welcome other explanations as we would generally expect underprediction bias in a large city like Dhaka for the reasons outlined above.

Together, these studies are complementary and address a critical gap in understanding global VMS distributions. In particular, the Guangzhou comparison demonstrates that developing industrial emissions inventories, particularly for D4, should be a priority for improving global VMS models. We thank the authors for making their data available and hope future researchers will leverage the model data (DOI: 10.25820/data.007521) to perform similar comparisons. Only through continuous model–observation comparisons can we advance our understanding of the global distribution of infrequently measured species such as VMS.

## References

[1] Xiao Y., Zhao S., Wang W., Asante K. A., Habib A., Bong C. W., Syed J. H., Bartilol S., Weber R., Jones K. C. (2026). Highly Volatile POPs in Urban Air across Asia and Africa: Dominance of
Volatile Methylsiloxanes. Environ. Sci. Technol..

[2] Brunet C. E., Mohammadi S., Roozitalab B., Gibson N. K., Fernandez R. P., Saiz-Lopez A., Hornbuckle K. C., Stanier C. O. (2026). Modeled Global Impacts of Chlorine Oxidation and Temperature
Dependence on the Atmospheric Lifetime and Concentrations of Volatile Methyl
Siloxanes. Environ. Sci. Technol..

[3] Fernandez R. P., Cuevas C. A., Villamayor J., Feinberg A., Kinnison D. E., Vitt F., Bossolasco A., Barrera J. A., Reynoso A., Tomazzeli O. G. (2025). Short-Lived Halogen Sources and Chemistry in the Community Earth
System Model v2 (CESM2-SLH). EGUsphere.

[4] Kashyap, S. Indonesia Beauty & Personal Care Market, Industry Trends, Challenges and Opportunities to 2030. 2024. https://www.kenresearch.com/industry-reports/indonesia-beauty-personal-care-market (accessed 2026-02-02).

[5] Jiwani S. S., Antiporta D. A. (2020). Inequalities in access to water and soap matter for the COVID-19
response in sub-Saharan Africa. International Journal for Equity in Health.

[6] Cai Z., Xie Q., Yang L., Yuan B., Wu G., Zhu Z., Wu L., Chang M., Wang X. (2023). A novel method for spatial allocation of volatile chemical products
emissions: A case study of the Pearl River Delta. Atmos. Environ..

[7] Cheng L., Chang Y., Yu H., Che X., Tan W., Zhu L. (2025). Atmospheric emerging pollutants of cyclic volatile methyl siloxanes
across the Yangtze River Delta region revealed by mobile
measurements. Chemosphere.

[8] Yang J., Duan K., Kang S., Shi P., Ji Z. (2017). Potential feedback between aerosols and meteorological conditions in a
heavy pollution event over the Tibetan Plateau and Indo-Gangetic
Plain. Climate Dynamics.

